# Study of the efficacy of the Hero program: Cross-national evidence

**DOI:** 10.1371/journal.pone.0238442

**Published:** 2020-09-04

**Authors:** Belén Mesurado, María E. Oñate, Lucas M. Rodriguez, Natalia Putrino, Paulina Guerra, Claudia E. Vanney

**Affiliations:** 1 Consejo Nacional de Investigaciones Científicas y Técnicas (CONICET), Buenos Aires, Argentina; 2 Universidad Austral, Pilar, Argentina; University of Sao Paulo Medical School, BRAZIL

## Abstract

The present study focuses on an analysis of the efficacy of the online intervention program called “Hero” for promoting prosociality and other socioemotional variables related to prosocial behavior, such as empathy, positive emotions, and forgiveness, in two Latin American countries: Argentina and Uruguay. The final Argentinean sample consisted of 579 adolescents (experimental group = 319 and control group = 260), and the Uruguayan sample consisted of 330 adolescents (experimental group = 140 and control group = 169), aged 12 to 15 years old. The ‘Hero’ program provided evidence of efficacy for the promotion of prosociality, empathy, positive emotions, and attitudes of forgiveness. It impacted each of the dimensions comprising these variables differently depending on the country where it was applied. We discuss the differences found in each country.

## Introduction

In recent years, the study of positive development in children and adolescents has gained great relevance. Positive development emphasizes the study and promotion of abilities, psychological resources, values and positive social relationships [1]. Numerous studies in adolescents have shown the benefits of engaging in prosocial behaviors [2, 3]. Hence, there is a need for tools or intervention programs that promote prosociality among adolescents in different areas of interpersonal development, such as within the family, friendships, and society in general.

There are different prosociality intervention programs that have proven effective. However, the majority are lengthy, involve face-to-face implementation and have been implemented only in the country in which they were developed [4]. It should be noted that the exception is the CEPIDEA program, which has been applied in at least three different countries: Italy [5, 6], Chile and Colombia [7]. The fact that an intervention program has been applied in different cities and countries ensures the reliability of its efficacy and indicates the program’s flexibility to adapt to different cultural contexts.

To our knowledge, the Hero program is the only short program with a virtual modality whose usability and efficacy in promoting prosocial behaviors has been studied [8]; however, these studies require significant methodological improvements to ensure the reliability of their results.

With an improved research design, the present study focused on analyzing the efficacy of the online intervention program called Hero for promoting prosociality and other socioemotional variables related to prosocial behavior, such as empathy, positive emotions (e.g., joy, gratitude), and forgiveness, in two Latin American countries: Argentina and Uruguay.

### Hero program description

The Hero program was developed in Argentina by Mesurado et al. in 2019 [8] and targets adolescents aged 12 to 15 years. Its objective is to promote prosociality through five socioemotional variables to make the effects of the intervention more lasting.

To our knowledge, this is the first virtual intervention program aimed at promoting prosocial behavior and the socioemotional variables linked to it [8, 9]. Several advantages have emerged from virtual modality programs. First, they are highly structured intervention programs. Second, self-administered programs could improve adolescents’ personal reflection and self-interest in topics. Third, guided virtual modality programs are more cost-effective than face-to-face intervention programs. Fourth, online programs are easily spread.

Hero is a self-administered program accessed from a Web page. To enter the program, adolescents must generate a username and choose an avatar, that is, they must select a virtual identity that represents them within the application. The program is guided by a Sensei who accompanies the adolescent throughout the application and presents the activities to be carried out through text and audio instruction, using the Spanish language with a neutral Latin American accent.

The program is structured in five linked modules for promoting empathy (understanding someone else’s emotional state) [10], gratitude (being aware of having received some type of personal benefit from someone) [11], positive emotions (emotional experiences in which pleasure or well-being predominate) [12], forgiveness (“the process by which thoughts, emotions, and negative behaviors toward an offender are transformed into thoughts, emotions and more positive prosocial thoughts”) [13], and finally, prosociality (voluntary behavior aimed at helping other person, such as strangers, friends, family members) [14]. According to Mesurado and colleagues (2019), the variables empathy, gratitude, positive emotions, and forgiveness were chosen to be part of the program because they can be taught and because there is empirical evidence showing their predictive effects on prosociality [8, 9].

The program is presented as an adventure that consists of a trip to five islands. The adolescent participates in the different phases in a sequential and predetermined way by visiting the island of empathy, the island of gratitude, the island of positive emotions, the island of forgiveness, and the island of prosocial behavior. The stay in each of the islands coincides with an intervention session, which lasts approximately 30 and 40 minutes. When the adolescent arrives on an island, he or she watches one episode of a psychoeducational video that deals with the behavior to be stimulated and then performs a series of activities. The videos narrate different conflictive situations in the daily lives of four adolescents (two females and two males). The conflict is resolved in each episode by exercising the socioemotional variable corresponding to the visited island. The videos offer a brief explanation of this variable and conclude with three brief suggestions from Sensei with exercises to stimulate it. The two or three activities that the adolescents then carry out are different on each island; some are playful, while others are reflective, relaxing, etc. (details of the activities for each island can be found in the papers by Mesurado and colleagues [8, 9].

### Preliminary usability studies of the Hero program

The usability of a program refers to the degree of satisfaction, efficiency and efficacy for the specific objectives that should be achieved by users of the application, in this case, adolescents. In recent studies, the creators of the program have conducted prefeasibility studies of usability, analyzing the level of acceptance, perceived usefulness, and participant satisfaction [8, 9].

Based on a sample of 51 adolescents from Argentina, the researchers analyzed the perceived ease of use of Hero and the possibility of transferring online activities to daily life [8]. They also inquired whether the adolescents would recommend the application to other users. The results showed that more than 80% of the adolescents included in the studies indicated that the Hero program was acceptable, easy to use, and useful and that they would be able to transfer the activities learned to daily life. In addition, 74% of the adolescents stated that they would recommend the application to other adolescents. The study also analyzed the efficacy of the Hero program for promoting prosocial behaviors, finding evidence of its ability to promote prosocial behavior toward strangers and family members but not toward friends.

The researchers also analyzed users’ opinions on the general characteristics of Hero, identifying the aspects of the program that were most pleasurable and/or unpleasurable among adolescents [9]. The adolescents who participated in the program gave it positive ratings for characteristics such as its format, creativity, originality, and the entertainment level of the activities. They also reported that the program activities helped them reflect on their own values and personal experiences. Most expressed that the psychoeducational videos were the activities they liked the most. Among the least liked aspects were the length and reiteration of the questions at the beginning and end of the intervention process to evaluate the variables, in addition to a few technical problems mentioned.

Although the outcomes of the preliminary usability studies for Hero were favorable, we feel that several methodological limitations prevent us from accepting the results as conclusive. First, the studies published to date did not compare the intervention group with a control group, which is key to ensuring that the effects are actually due to the intervention and are not a result of developmental changes among the adolescents. Second, since no follow-up measurements were performed, it was not possible to determine whether the changes in prosocial behavior observed at the end of the program were maintained over time. We also noted that the studies carried out to date did not consider certain factors that could affect the efficacy of the program, such as cultural differences among participants from different countries. Finally, given the characteristics of the program, it is likely that Hero is also effective for promoting other socioemotional variables associated with prosociality, such as empathy, positive emotions, and attitudes of forgiveness. However, this has not been tested empirically through assessment scales.

### The present study

To remedy the aforementioned weaknesses, this study includes a comparison between an intervention group and a control group, in addition to performing a follow-up measurement. Additionally, given that there have been numerous studies showing that women score higher on measures of prosocial behavior [15, 16]; empathy [15, 17]; positive emotions, specifically gratitude [18, 19]; and forgiveness [20], in the present study, the influence of gender differences is studied.

Furthermore, to analyze the flexibility of the Hero program to adapt to different cultural contexts, research will be conducted in two countries. Argentina and Uruguay were chosen as the countries in which to conduct the research because, despite their geographic proximity and the common use of the Spanish language, the characteristics of these countries indicate a certain cultural and sociodemographic distance between them.

#### Sociodemographic characteristics of Argentina and Uruguay

Uruguay and Argentina are neighboring countries that, despite geographical proximity, have their differences. In the words of Borges [21], these countries present “just a to-and-fro in proximity and distance”. Historically, their territories were occupied by the same aboriginal groups [22, 23], and after Spanish colonization, they were part of the Viceroyalty of the Río de la Plata [24]. With the uprisings of 1815 and 1825, “La Banda Oriental”, Uruguay began to differentiate itself from Argentina, and in 1830, it established its own constitution and began to be called the “Eastern Republic of Uruguay” [25, 26]. That is, Uruguay and Argentina gained independence as separate countries only 190 years ago.

In terms of territory, Uruguay is the second smallest country in Latin America after Suriname [27] and has a striking demographic characteristic: it has maintained approximately three million inhabitants for more than 30 years [28]. Argentina’s territory is 16 times larger than Uruguay’s, and its population is 15 times larger [29]

According to data from the World Bank [29–31] for 20 Latin American countries, Argentina is the country with the third-highest public debt, as calculated by percentage, and Uruguay has the fourth highest. Uruguay is the country with the highest per capita income in the region, while Argentina ranks fifth. According to the World Bank [32], Uruguay stands out from the rest of the Latin American countries due to its high per capita income level; the Uruguayan middle class represents 60% of the population, with poverty being quite low and destitution almost nil, and it offers a high level of equal opportunity for access to basic services. In Uruguay, corruption rates are low, and citizens trust their governments. In contrast, while Argentina is a country noted for its abundant natural resources and is one of the largest economies in the region due to its GDP, the instability in its growth and considerable institutional obstacles have impeded its development. Poverty is high, and child poverty in particular is very high, as one in two Argentine children up to 14 years of age is poor.

Concerning educational levels, Argentina and Uruguay have a similar share of the population 25 years old and older by educational attainment. According to the UNESCO Institute for Statistics (UIS) in Argentina, the 35.5% of those older than 25 years old completed primary education, 37.2% completed secondary education, and 20% completed Bachelor's or equivalent educations; and in Uruguay, 34.4% completed primary education, 43.5% completed secondary education, and 13.3% completed Bachelor's or equivalent educations [33]. In 2018, Argentina had approximately 2.5 million students enrolled in lower secondary general education (adolescents aged 12 to 15 years old), while in 2017, Uruguay had approximately 150,000 students [33].

Regarding religious practice, Uruguay has the lowest percentage of Catholics (38%) and the highest percentage of atheists or nonreligious people in Latin America (41%) [34, 35]. In contrast, in Argentina, approximately 76% identify as Catholic, and 11% are indifferent to religion, that is, agnostic, atheist or without religion [36, 37]. Regarding the importance of religion, in 2011, approximately 60% of Uruguayans indicated that they considered religion unimportant, while in 2013, approximately 60% of Argentines indicated that they considered it important [38].

According to the World Values Survey (2010–2014), 87.3% of Argentineans and 86.3% of Uruguayans younger than 29 years old believe that family is very important in their lives. Similar patterns are found in both countries when analyzing the importance attributed to friendship, with percentages of 66% and 61% in Argentina and Uruguay, respectively. In contrast, there are important differences between the two countries when attitudes toward strangers are analyzed. In Argentina, approximately 57% of young people believe that it is important to show tolerance and respect for other people, while in Uruguay, 87% express this belief [38]. Moreover, 90% of Argentineans believe that immigration is negative for the growth and development of the country [39], while only 31% of Uruguayans believe it [40].

#### Objectives of this new research

The objectives of this new research can be summarized as follows: we propose to analyze the efficacy of the Hero program for the promotion of prosociality, empathy, positive emotions (e.g., joy, gratitude), and forgiveness. Specifically, the effect of the Hero program will be evaluated at a three-month follow-up period and compared to a control group in two Latin American countries: Argentina and Uruguay. The gender of the participants will be analyzed to determine whether gender had an effect on the efficacy of the program.

## Materials and methods

The study and procedures were approved by the Institutional review board at Universidad Austral [CIE 19–031]. The data were analyzed anonymously.

### Research design

Five secondary schools with 31 classrooms in Argentina and four schools with 19 classrooms in Uruguay were intentionally selected to participate in the study. We carried out a cluster randomized trial at each school [41, 42]. We randomized classrooms, rather than schools, because we expected having adolescents with similar patterns in the experimental and control groups. All of the schools included in the study are privately run. For inclusion, we required that the adolescents be 12 to 15 years old and not be participating in another intervention program. The students who comprised the control group were placed on a waiting list to participate in the Hero program once the follow-up evaluation was completed. Both the experimental and control groups participated in three pretest evaluations (before the start of the intervention), a posttest evaluation (one week after the end of the intervention program) and a follow-up evaluation (10 weeks after completing the posttest evaluation).

### Participants

#### Sample from Argentina

The initial sample consisted of 778 participants from two important provinces in Argentina: Entre Ríos and Buenos Aires. The sample was collected from urban areas.

**Fig 1:**
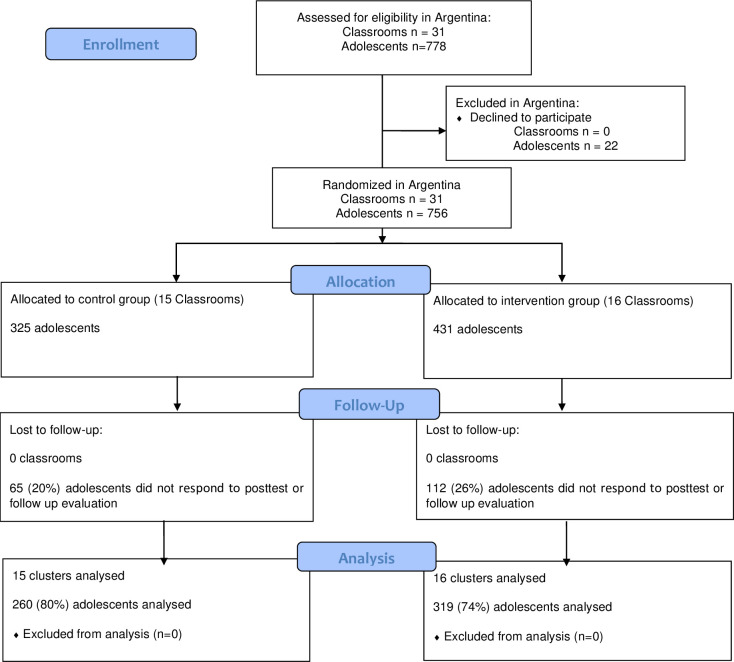
Argentinean sample flow diagram following the Consolidated Standards of Reporting Trials (CONSORT) recommendations.

Fig 1 shows the Argentinean sample flow diagram following the Consolidated Standards of Reporting Trials (CONSORT) recommendations [41, 42]. The final sample of the experimental group consisted of 319 adolescents aged 12 to 15 years (M = 13.64, SD = .95), and 53% of the participants were female. The control group consisted of 260 adolescents aged 12 to 15 years (M = 13.22, SD = .97), 53% of whom were female.

#### Sample from Uruguay

The initial sample consisted of 396 participants from two cities in Uruguay -Salto and Belén- which belong to the Salto department (second most important department after Montevideo). The sample was collected from urban areas.

**Fig 2:**
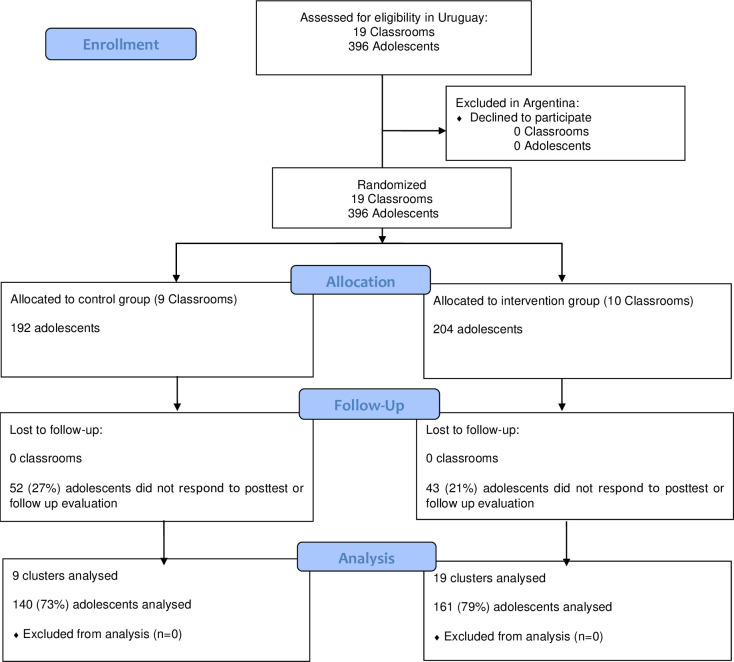
Uruguayan sample flow diagram following the Consolidated Standards of Reporting Trials (CONSORT) recommendations.

Fig 2 shows the Uruguayan sample flow diagram following CONSORT recommendations [41, 42]. The final sample of the experimental group consisted of 161 adolescents aged 12 to 15 years (M = 13.73, SD = .92), and 35% were female. The control group consisted of 140 adolescents aged 12 to 15 years (M = 13.24, SD = .93), 38% of whom were female.

### Procedure

We communicated with the authorities of the educational institutions to explain the objective of the Hero program and its characteristics (number of sessions, method of application, technological requirements, etc.). Once authorizations from the educational institutions were obtained, a letter was sent to each of the parents or guardians of the students aged 12 to 15 years telling them about the research project and asking for their authorization for their child to participate. The parents or guardians of the adolescents signed an informed consent form. Only adolescents who agreed to participate and who had the consent of their parents were included in the study.

Once the authorizations were obtained, classes at each school were chosen randomly to participate in the experimental group or the waitlisted control group. The students used the computer room of the educational institution and participated in the eight sessions of the Hero program during school hours at the times granted by the authorities of the institution.

Each session was directed by a psychologist trained in the use of the program, who ensured the proper functioning of the computer application and directed the adolescents on the tasks when necessary. Throughout the implementation of the program, the research team also had computer technical support.

The participants in the experimental group participated in eight sessions: 1. Pretest evaluation; 2. Empathy session; 3. Gratitude session; 4. Positive emotions session; 5. Forgiveness session; 6. Prosocial behavior session; 7. Posttest evaluation; and 8. Follow-up evaluation. The control group participated in the pretest, posttest, and follow-up evaluations. Each session lasted approximately 30–40 minutes. The adolescents did not receive any compensation for participating in the research project.

### Evaluation instruments

Participants from both countries in both the experimental group and the control group completed the following assessment tools during the pretest, posttest, and follow-up assessments. The instrument evaluation was blinded for the participants.

#### Prosociality

To evaluate prosociality, a modified version of the Kindness and Generosity subscale extracted from the Values in Action Inventory of Strengths [43] by Padilla-Walker and Christensen [44] was used. This questionnaire evaluates prosocial behavior directed toward strangers, friends and family members and was adapted to the Spanish language by Mesurado, Guerra [45].

The questionnaire comprises 27 items, which the participants answered using a five-point scale ranging from 1 (“not like me at all”) to 5 (“very much like me”). The items are distributed in three subscales, one for each target toward which the behavior is directed, namely, nine items to evaluate prosocial behavior toward strangers (e.g., “I help people I do not know even if it is not easy for me”), nine items for prosocial behavior toward friends (e.g., “I always listen to my friends talk about their problems”) and nine items for prosocial behavior toward the family (e.g., “I help my family even if it is not easy for me”). The score for each dimension is obtained from the average of the scores for each item.

The instrument showed good psychometric properties of reliability and validity, both in its original version [44] and in the Spanish version [45]. The reliability indices obtained in this study for the Argentina and Uruguay samples are shown in Table 1.

**Table 1:** Reliability of the scales.

Variables	McDonald’s coefficient omega Pretest		McDonald’s coefficient omega Posttest		McDonald’s coefficient omega Follow up	
	Argentina	Uruguay	Argentina	Uruguay	Argentina	Uruguay
**Prosocial behavior**						
Strangers	.87	.92	.85	.91	.78	.84
Friends	.93	.95	.93	.95	.89	.94
Family	.93	.95	.94	.94	.90	.93
**Empathy**						
Emotional Regulation	.86	.85	.82	.91	.80	.88
Emotional Contagion	.80	.84	.80	.89	.82	.85
Perspective Taking	.86	.89	.82	.91	.79	.86
Emotional Awareness	.86	.89	.83	.91	.81	.86
Empathic Action	.84	.86	.82	.89	.88	.89
**Positive Emotions**						
Joy	.71	.82	.77	.80	.79	.80
Gratitude	.79	.79	.76	.74	.74	.77
Serenity	.86	.72	.82	.82	.85	.81
Satisfaction	.85	.82	.82	.75	.76	.75
Sympathy	.73	.83	.81	.82	.77	.81
**Forgiveness**						
Absence of negative emotions	.85	.83	.86	.88	.84	.89
Presence of positive emotions	.85	.90	.86	.84	.79	.83

#### Empathy

To evaluate empathy, the Empathy Questionnaire by Richaud, Lemos [46] was used. The questionnaire consists of 15 items with four response options: 1 = never, 2 = sometimes, 3 = often and 4 = always. It evaluates five aspects of empathy: emotional contagion (feeling other people’s emotions and behaviors; three items, e.g., “When I see someone dancing, I feel like moving my feet”), emotional awareness (being able to recognize the emotions of others; three items, e.g., “Even though I am happy, I notice when a friend is angry”), perspective taking (being able to understand another's point of view; three items, e.g., “When I argue with someone, I try to understand what he or she is thinking”), emotional regulation (ability to manage one’s own emotions; three inversely stated items, e.g., “When I get angry, I find it difficult to calm down”) and empathic action (consolidating empathic emotion into a specific action; three items, e.g., “We must share with those who have less than us”). The instrument showed good psychometric properties of validity and reliability in its original version [46].

#### Positive emotions

Positive emotions are emotional experiences in which pleasure or well-being predominate. These emotions were evaluated using the Positive Emotions Questionnaire developed by Oros [47]. The questionnaire consists of 23 items that operationalize five positive emotions: (a) joy, (b) gratitude, (c) sympathy, (d) personal satisfaction, and (e) serenity. The items are written affirmatively, briefly (less than 12 words) and in the first person, and each describes a single experience or behavior. Depending on the extent to which the person agrees with each statement, he or she can choose from three possible responses: 1 = no, 2 = somewhat, or 3 = yes.

Examples of the items are as follows: for the joy dimension, “I am almost always happy”; for the gratitude dimension, “I like to thank people”; for the serenity dimension, “I am almost always relaxed”; for the sympathy dimension, “I feel very badly if I see someone getting hurt”; and for the personal satisfaction dimension, “I feel like I am very valuable”.

#### Forgiveness

The capacity to forgive was evaluated with The Forgiveness Scale by Rye, Loiacono [48], which measures forgiveness of a specific offender.

The scale contains 15 items (eight are inversely stated) using the phrase *“Think of how you have responded to the person who has wronged or mistreated you”*. Two aspects of forgiveness are evaluated: the absence of negative emotions toward the aggressor (e.g., “I have been able to let go of my anger toward the person who wronged me”; inversely stated, “I cannot stop thinking about how I was wronged by this person”) and the presence of positive emotions toward the aggressor (e.g., “I wish for good things to happen to the person who wronged me”). The items are rated on a Likert-type scale ranging from 1 (strongly disagree) to 5 (strongly agree). Higher scores on this scale reflect a greater degree of forgiveness toward the aggressor. Rye, Loiacono [48] demonstrated the good psychometric properties of the scale, showing that it possessed adequate reliability through internal consistency and stability and proving its adequate validity by analyzing its factorial, convergent, and construct validity.

### Statistical procedure

The means and standard deviation of each of the dimensions of the variables included in the study were calculated for both countries (see Tables 2 and 3). Multivariate analysis of variance (MANOVA) was used to evaluate the efficacy of the program (experimental group vs. control group) and the effect of gender (female and male) and to determine the effects on prosociality, empathy, positive emotions, and forgiveness in each country.

**Table 2:** Mean and standard deviation values of variables across pretest, posttest and follow up evaluation in Argentinean sample.

Variables	Intervention group						Control group					
	Pretest		Posttest		Follow up		Pretest		Posttest		Follow up	
	M	SD	M	SD	M	SD	M	SD	M	SD	M	SD
**Prosocial behavior**												
Strangers	3.01	.04	3.22	.04	3.11	.04	3.09	.04	3.07	.05	3.09	.05
Friends	4.03	.04	4.16	.04	4.39	.04	4.39	.05	4.23	.05	4.22	.04
Family	3.95	.05	4.04	.05	4.12	.04	4.04	.05	3.97	.05	3.91	.04
**Empathy**												
Emotional Regulation	2.49	.04	2.48	.04	2.53	.04	2.59	.05	2.65	.05	2.70	.05
Emotional Contagion	2.29	.04	2.53	.04	2.55	.04	2.15	.04	2.11	.05	2.17	.05
Perspective Taking	2.93	.04	2.92	.04	2.90	.04	2.92	.04	2.90	.04	2.86	.04
Emotional Awareness	3.06	.04	3.16	.04	3.13	.03	3.17	.04	3.10	.04	3.11	.04
Empathic Action	3.15	.04	3.22	.04	3.29	.04	3.38	.05	3.29	.05	3.22	.04
**Positive Emotions**												
Joy	2.59	.03	2.60	.03	2.58	.03	2.51	.03	2.51	.03	2.52	.03
Gratitude	2.79	.02	2.72	.02	2.65	.02	2.78	.02	2.72	.02	2.71	.03
Serenity	2.27	.03	2.38	.03	2.37	.03	2.21	.03	2.24	.03	2.27	.03
Satisfaction	2.30	.04	2.34	.04	2.32	.04	2.23	.04	2.23	.04	2.22	.04
Sympathy	2.34	.03	2.34	.03	2.32	.03	2.28	.03	2.27	.03	2.27	.03
**Forgiveness**												
Absence of negative emotions	3.19	.04	3.22	.04	3.39	.04	3.44	.04	3.53	.04	3.57	.04
Presence of positive emotions	2.62	.04	2.78	.05	2.74	.05	2.70	.05	2.67	.05	2.64	.05

**Table 3:** Mean and standard deviation values of variables across pretest, posttest and follow up evaluation in Uruguayan sample.

Variables	Intervention group						Control group					
	Pre-test		Posttest		Follow up		Pretest		Posttest		Follow up	
	M	SD	M	SD	M	SD	M	SD	M	SD	M	SD
**Prosocial behavior**												
Strangers	3.14	.07	3.41	.07	3.51	.07	2.97	.07	2.82	.08	2.63	.07
Friends	4.03	.07	4.10	.08	4.08	.07	3.92	.07	3.74	.08	3.63	.08
Family	4.14	.07	4.16	.07	4.15	.07	4.01	.07	3.84	.08	3.68	.08
**Empathy**												
Emotional Regulation	2.74	.07	2.68	.07	2.87	.07	2.63	.07	2.69	.08	2.64	.07
Emotional Contagion	2.56	.06	2.71	.07	2.77	.06	2.35	.06	2.26	.08	2.18	.07
Perspective Taking	2.66	.06	2.77	.06	3.02	.06	2.61	.06	2.50	.07	2.57	.06
Emotional Awareness	3.08	.05	3.11	.06	3.16	.06	3.07	.06	2.97	.06	2.93	.06
Empathic Action	3.50	.06	3.41	.06	3.47	.06	3.32	.06	3.24	.07	3.11	.06
**Positive Emotions**												
Joy	2.69	.04	2.66	.04	2.71	.03	2.61	.04	2.60	.04	2.53	.04
Gratitude	2.85	.03	2.81	.03	2.81	.03	2.79	.03	2.71	.03	2.69	.03
Serenity	2.54	.04	2.57	.04	2.60	.04	2.47	.04	2.44	.04	2.39	.04
Satisfaction	2.53	.05	2.53	.05	2.61	.05	2.47	.05	2.45	.05	2.38	.05
Sympathy	2.43	.04	2.47	.04	2.47	.04	2.37	.04	2.34	.05	2.29	.04
**Forgiveness**												
Absence of negative emotions	3.21	.05	3.19	.06	3.49	.07	3.30	.05	3.44	.06	3.41	.07
Presence of positive emotions	3.05	.08	3.13	.08	3.33	.08	2.91	.08	2.83	.09	2.68	.08

## Results

To examine the effects of the Hero program, we carried out four 2 (intervention: experimental group and control group) x 2 (gender: male and female) x 3 (time: pretest, posttest, and follow-up) MANOVAs with repeated measures for prosocial behavior, empathy, positive emotions, and forgiveness. Below, we present the results for each variable in the Argentinean and Uruguayan samples. The MANOVAs were performed using SPSS software. Tukey’s post hoc test for the interaction effect of time and intervention was performed using STATISTICA software.

### Efficacy of the Hero program for promoting prosocial behavior toward strangers, friends and family

The main effects of intervention, gender, and time were significant in both countries. Moreover, the interaction effect of time and intervention was significant, although the interaction effect of time and gender was not significant in either country. Finally, the interaction effect of time, condition, and gender was significant only in Argentina (see Table 4).

**Table 4:** Efficacy of the Hero program for promoting prosocial behavior, empathy, positive emotions, and forgiveness in Argentina and Uruguay.

	Argentina					Uruguay				
Prosocial behavior	Wilk’s λ	F	df	*p*	eta	Wilk’s λ	F	df	*p*	eta
Intervention	0.98	4.30	3, 573	.01	.02	0.87	14.54	3, 295	.001	.13
Gender	0.85	33.81	3, 573	.001	.15	0.92	8.04	3, 295	.001	.08
Time	0.94	5.56	6, 570	.001	.06	0.95	2.54	6, 292	.05	.05
Time x Intervention	0.85	15.59	6, 570	.001	.14	0.86	7.93	6, 292	.001	.14
Time x Gender	0.99	1.31	6, 570	.25	-	0.98	0.88	6, 292	.51	-
Time x Intervention x Gender	0.98	2.44	6, 570	.05	.03	0.98	0.77	6, 292	.59	-
**Empathy**										
Intervention	0.92	10.49	5, 571	.001	.08	0.89	7.34	5, 293	.001	.11
Gender	0.77	34.05	5, 571	.001	.23	0.90	6.63	5, 293	.001	.10
Time	0.94	3.35	10, 566	.001	.06	0.90	3.35	10, 288	.001	.10
Time x Intervention	0.91	5.77	10, 566	.001	.09	0.90	3.28	10, 288	.001	.10
Time x Gender	0.97	1.5	10, 566	.13	-	0.97	0.98	10, 288	.46	-
Time x Intervention x Gender	0.96	2.26	10, 566	.05	.04	0.96	1.97	10, 288	.29	-
**Positive Emotions**										
Intervention	0.96	4.365	5, 565	.001	.04	0.96	2.62	5, 293	.05	.04
Gender	0.79	30.01	5, 565	.001	.21	0.84	11.10	5, 293	.001	.16
Time	0.86	9.08	10, 560	.001	.14	0.95	1.52	10, 288	.13	-
Time x Intervention	0.98	1.09	10, 560	.37	-	0.95	1.56	10, 288	.12	-
Time x Gender	0.98	1.02	10, 560	.42	-	0.95	1.35	10, 288	.21	-
Time x Intervention x Gender	0.99	.40	10, 560	.95	-	0.94	1.96	10, 288	.04	.06
**Forgiveness**										
Intervention	0.95	15.95	2, 572	.001	.05	0.94	9.54	2, 296	.001	.06
Gender	0.98	6.00	2, 572	.01	.02	0.97	5.30	2, 296	.01	.04
Time	0.93	10.46	4, 570	.001	.07	0.95	4.08	4, 296	.01	.05
Time x Intervention	0.98	3.12	4, 570	.001	.02	0.92	6.55	4, 294	.001	.08
Time x Gender	0.99	1.51	4, 570	.20	-	0.99	0.16	4, 294	.96	-
Time x Intervention x Gender	0.97	3.70	4, 570	.01	.03	0.99	0.13	4, 294	.97	-

In both countries, the adolescents who participated in the intervention program showed greater prosocial behavior toward strangers [Argentina F(2, 1150) = 4.40, *p <* .01, eta = .01; Uruguay F(2, 594) = 24.31, *p <* .001, eta = .08], friends [Argentina F(2, 1150) = 38.15, *p <* .001, eta = .06; Uruguay F(2, 594) = 5.61, *p <* .01, eta = .02], and family members [Argentina F(2, 1150) = 11.58, *p <* .001, eta = .02; Uruguay F(2, 594) = 5.48, *p <* .01, eta = .02] than the participants in the control group at the different evaluation times.

In Argentina, the effect of the interventions on prosocial behavior toward friends and family remained stable (difference between follow-up and pretest Tukey’s post hoc test t *p <* .001), with the exception of prosocial behavior toward strangers, which decreased in the follow-up evaluation (see Table 2).

In the case of Uruguay, the effect of the interventions on prosocial behavior toward strangers remained stable (difference between follow-up and pretest Tukey’s post hoc test t *p <* .001); see Table 3. In addition, prosocial behavior toward friends and family significantly decreased in the control group (difference between follow-up and pretest Tukey’s post hoc t *p <* .001) but remained stable in the intervention group (see Table 3).

### Efficacy of the Hero program for promoting different aspects of empathy

The main effects of intervention, gender, and time were significant in both countries. Moreover, the interaction effect of time and intervention was significant, although the interaction effect of time and gender was not significant in either country. Finally, the interaction effect of time, condition, and gender was significant only in Argentina; see Table 4.

The experimental group showed higher levels of emotional contagion than the control group participants in both countries [Argentina F(2, 1150) = 12.25, *p <* .001, eta = .02; Uruguay F(2, 594) = 8.53, *p <* .001, eta = .03]. Moreover, in Argentina, the experimental group showed higher levels of emotional awareness [F(2, 1150) = 5.31, *p <* .01, eta = .06] and empathic action [F(2, 1150) = 11.34, *p <* .001, eta = .02] than the control group participants at the different evaluation times, but no differences in emotional regulation and perspective taking were found. In Uruguay, the experimental group participants had higher levels of perspective taking than the control group participants did [F(2, 594) = 9.62, *p <* .001, eta = .03] at the different evaluation times, but no differences in emotional regulation, emotional awareness, and empathic action were found.

In Argentina, the effect of the interventions on emotional contagion and empathic action remained stable in the follow-up evaluation (difference between follow-up and pretest for emotional contagion Tukey’s post hoc test t *p <* .001 and for empathic action Tukey’s post hoc test t *p <* .01). In the intervention group, emotional awareness initially increased but then decreased in the follow-up evaluation (difference between follow-up and pretest Tukey’s post hoc test t *p* = .32); see Table 2.

In the case of Uruguay, the effect of the interventions on emotional contagion and perspective taking remained stable (difference between follow-up and pretest Tukey’s post hoc test t *p <* .001); see Table 3.

### Efficacy of the Hero program for promoting positive emotions

The main effects of intervention and gender were significant in both countries, while the main effects of time were significant in Argentina. Finally, the interaction effect of time, condition, and gender was significant only in Uruguay; see Table 4.

In Argentina, the intervention increased participants’ serenity [F(2, 1138) = 2.20, *p <* .001, eta = .02] and remained stable (difference between follow-up and pretest Tukey’s post hoc test t *p <* .001); see Table 2. Concerning Uruguay, participants in the experimental group had higher levels of joy [F(2, 594) = 2.92, *p <* .05, eta = .01], serenity [F(2, 594) = 3.22, *p <* .05, eta = .01] and satisfaction [F(2, 594) = 4.47, *p <* .01, eta = .02] than the participants in the control group did at the different evaluation times.

### Efficacy of the Hero program for promoting forgiveness

The main effects of intervention, gender, and time were significant in both countries. Moreover, the interaction effect of time and intervention was significant, although the interaction effect of time and gender was not significant in either country. Finally, the interaction effect of time, condition, and gender was significant only in Argentina; see Table 4.

The participants in the experimental group had higher levels of the presence of positive emotions toward an aggressor than the participants in the control group did in both countries [Argentina F(2, 1146) = 4.16, *p <* .05, eta = .01; Uruguay F(2, 594) = 9.36, *p <* .001, eta = .03]. Moreover, in Uruguay, the participants in the experimental group had higher levels of the absence of negative emotions toward an aggressor than the control group [F(2, 594) = 5.98, *p <* .01, eta = .02].

In Argentina, the effect of the interventions on the presence of positive emotions toward an aggressor initially increased but then decreased in the follow-up evaluation (difference between follow-up and pretest Tukey’s post hoc test t *p* = .13); see Table 2. In the case of Uruguay, the effect of the interventions on the presence of positive emotions toward an aggressor (Tukey’s post hoc test t *p <* .001) and the absence of negative emotions toward an aggressor remained stable (difference between follow-up and pretest Tukey’s post hoc test t *p <* .01); see Table 3.

## Discussion

As mentioned, previous studies of the efficacy of the Hero program have important limitations. First, in-depth studies of the program were not developed to analyze the program’s efficacy by comparing its results with a control group. Second, whether its efficacy for the promotion of prosocial behaviors remained stable over time was not analyzed. Our study sought to remedy these limitations and study whether the Hero program is effective in different countries. To achieve these objectives, improvements in the research design were incorporated, and the study was expanded to include two groups of adolescents from different countries: Argentina and Uruguay.

The analysis of the efficacy of the program, which included the comparison of the three evaluation periods, found that the Hero program was effective for promoting prosocial behavior in adolescents from Argentina and Uruguay. In both countries, the adolescents who participated in the intervention program showed greater prosocial behavior toward strangers, friends, and family members than the participants in the control group. These results show the important role played by the program in promoting prosociality among adolescents in both countries.

In addition, the results for the efficacy of the program for the promotion of prosociality in both countries agreed with the cultural similarities. In fact, previous national surveys have found that Argentines attribute similar importance to friendships and meet daily or weekly with their friends like Uruguayans did; moreover, the importance given to family does not vary from one country to the other [38].

However, we also found some differences between the two countries that we did not expect. In all cases, the effects of the program on prosociality were constant over time, with the exception of prosocial behavior toward strangers in the Argentine adolescents, which showed a decline during the follow-up evaluation. This difference could be explained by Uruguayan adolescents being stimulated by parents to be more tolerant and respectful with strangers than Argentinian parents do [38]. It is probable that the Hero program strengthens ideas and concepts previously learned; consequently, the effects of the program toward strangers remained stable. In the Argentinean case, it might be necessary to stimulate for more time the prosocial behavior toward strangers to obtain more stable effects because it is necessary modifying more deeply rooted cultural characteristics.

Regarding gender, although we found an effect of gender on the three types of prosociality in both countries that indicated that females presented higher levels of prosociality toward the three targets (strangers, friends and family) than males, we found no effect in the interaction between gender and the intervention, which indicates that the program was equally effective in females as in males.

Given that empathy is a multidimensional construct that is intimately related to prosocial behavior, we analyzed the efficacy of the Hero program for promoting empathy in adolescents. The results indicated that the program was effective for promoting empathy in both countries. Specifically, when analyzing the interaction of the intervention and time, we found that, in Argentina, the adolescents in the experimental group reported higher levels of emotional contagion (i.e., feeling the same emotion that another person is experiencing), empathic action (which refers to the emergence of helping behavior), and emotional awareness (referring to the ability to easily identify the emotions of others) than the control group. The adolescents in the Uruguayan experimental group reported higher levels of emotional contagion and perspective taking (the ability to understand the point of view of another) than the control group. These results provide evidence that the Hero program stimulates the two most important aspects of empathy: the emotional aspect (evaluated in this study through emotional contagion) and more cognitive aspects (such as perspective taking in the Uruguay sample or emotional awareness in the sample from Argentina). Finally, we found a gender effect on empathy in both countries that indicated that females have higher levels of emotional contagion, emotional awareness, perspective taking, and empathic action than males, while males experience a higher level of emotional regulation than females. However, we found no effect on the interaction of gender with the intervention, which indicates that the program was equally effective for females as for males.

In relation to positive emotions, the Hero program was shown to be effective, although only for promoting serenity in the Argentina sample. In the Uruguayan sample, adolescents who participated in the program had higher levels of serenity, joy, and satisfaction than the adolescents in the control group. The results indicate that among the positive emotions evaluated in this study, the serenity dimension was the one most stimulated by Hero. These results may have occurred because serenity was directly stimulated in the virtual environment of Hero, while other dimensions, such as joy and satisfaction, were indirectly promoted. In comparison, it is surprising that the program was not effective for promoting gratitude since a specific module of the program is targeted at stimulating this positive emotion. This could be because the instrument we used to evaluate gratitude mainly operationalizes the feeling of enjoyment derived from returning favors (e.g., I like to return favors) and reciprocity in the face of a supportive event (e.g., Whenever I can, I return the favors I receive) and does not evaluate broader attitudes of gratitude toward life or people, aspects that are probably more related to the activities promoted by Hero. Consequently, this fact will need to be studied in greater depth in future research.

Finally, the Hero program was effective for promoting attitudes of forgiveness against an aggressor in Argentinian and Uruguayan adolescents. Specifically, when comparing the differences between the experimental and control groups, we found that, in the Uruguayan sample, the adolescents who participated in the program had higher levels of positive emotions and a greater absence of negative feelings toward the aggressor than the adolescents in the control group did. In the case of Argentina, in contrast, there was a difference in positive emotions between groups, but the effect of the intervention did not maintain stability in follow-up evaluations. There was a gender effect on forgiveness in both countries that indicated that girls have fewer negative emotions and more positive emotions toward an aggressor than boys. However, we found no effect on the interaction of gender with the intervention, which would suggest that the program was equally effective for females as for males. In conclusion, this research provided deep evidence for the Hero program’s effectiveness. Because Hero is a brief online program, is self-administered, has a low cost, and requires a simple infrastructure (internet connection, computer, camera, and microphone), it could be easily disseminated as a possible solution for the promotion of socioemotional variables. Consequently, Hero could be scaled up in several school contexts and other educational institutions in Latin America.

### Limitations and future studies

In summary, while the Hero program provides evidence of efficacy for the promotion of prosociality, empathy, positive emotions, and attitudes of forgiveness, it impacts each of the dimensions comprising these variables differently depending on the sociocultural context in which it is applied. Consequently, it would be interesting to implement this program in a greater number of countries or cultural contexts to elucidate its efficacy with greater precision. On the other hand, it will also be important to include in future studies a more specific measurement of gratitude to allow a more detailed analysis of the effect of the program on different aspects of this variable.

Finally, although the Hero program was initially developed for adolescents in the general population, in the future, it could be extremely interesting to analyze its effectiveness in contexts of violence, in adolescents with difficulties in social interactions, and/or in poverty contexts.
