# Correction: Study of the efficacy of the Hero program: Cross-national evidence

**DOI:** 10.1371/journal.pone.0250287

**Published:** 2021-04-12

**Authors:** Belén Mesurado, María E. Oñate, Lucas M. Rodriguez, Natalia Putrino, Paulina Guerra, Claudia E. Vanney

In the Materials and methods section, there is an error in the first sentence of the first paragraph. The correct sentence is: The study and procedures were approved by the Institutional review board at Centro Interamericano de Investigaciones Psicológicas y Ciencias Afines (CIIPCA) [19–03].
